# Lambda light chain - restricted non - crystalline proximal tubulopathy with cast nephropathy in multiple myeloma: a case report and literature review

**DOI:** 10.1186/s12882-024-03721-9

**Published:** 2024-09-30

**Authors:** Mingfu Lan, Yaohui Guo, Caiyun Wang, Xiaoqin Wang, Jing Li, Yanxia Wang

**Affiliations:** 1 Departments of Pathology, Xijing Hospital, School of Basic Medicine, Fourth Military Medical University, 169 Changle Western Street, Xi’an, 710032 Shaanxi China; 2 School of Basic Medicine, Fourth Military Medical University, Xi’an, 710032 Shaanxi China

**Keywords:** Multiple myeloma, Light chain proximal tubulopathy, Light chain cast nephropathy, Acute kidney injury

## Abstract

**Background:**

Multiple myeloma (MM) often causes renal tubular damage, such as the light chain cast nephropathy (LCCN) and the light chain proximal tubulopathy (LCPT). The excessive light chains deposited in the proximal and distal tubules usually manifest with different characteristics, leading to a rare coexistence of the two pathological conditions. Here we report a unique case of a patient with multiple myeloma (MM) who presented with acute kidney injury (AKI) due to dual conditions of λ light chain-restricted non-crystalline LCPT and LCCN. This report reviews the clinical presentation and histological findings, comparing them with previously published cases.

**Case presentation:**

A 49-year-old male patient was admitted with a chief complaint of “fatigue, loss of appetite for 40 days and elevated blood creatinine for 10 days.” In serum and urine, the λ light chain level and the ratio of κ to λ free light chain were 1235 mg/dl and 93.25 mg/dl, 0.0022 and 0.0316, respectively. Additionally, serum protein electrophoresis showed an M-spike with monoclonal IgD-λ. Bone marrow puncture revealed 30.5% primitive naive plasma cells, indicative of IgD-λ MM. Light microscopy of kidney biopsy specimen showed periodic acid-Schiff (PAS)-negative cytoplasm in some proximal tubules and PAS-negative casts with a rigid appearance in some distal tubule lumens. On immunofluorescence, these proximal tubular epithelial cells cytoplasm and casts stained exclusively with λ-light chains. Electron microscopy did not reveal any crystalline inclusions. Given the clinical and bone marrow puncture findings, the overall pathological presentation was LCPT with LCCN secondary to IgD-λ MM. After chemotherapy and dialysis, the patient’s condition was improved and he was tracked in follow-ups.

**Conclusion:**

In some tubular renal injuries caused by MM, the morphological changes are subtle and often overlooked. In this paper, we present a rare case of LCPT with LCCN showing λ restriction in patient with MM. Through the clinicopathological analysis of patients, the understanding of the disease can be deepened and the diagnosis rate improved.

**Supplementary Information:**

The online version contains supplementary material available at 10.1186/s12882-024-03721-9.

## Background

Multiple myeloma (MM) is caused by clonal proliferation of plasma cells that produce monoclonal immunoglobulins or light chains. This proliferation often leads to damage to associated organ or tissue. MM causes different types of renal injury, and some renal histomorphological changes in MM are subtle and often overlooked by the unwary, especially in renal tubular damage such as LCPT.

LCCN is the most common pathological manifestation of MM, characterized by the accumulation of monotypic light chains within the distal renal tubules. These light chains bind to THP to form casts, which often manifest as acute kidney injury (AKI). LCPT is a rare pathological manifestation of monoclonal gammopathy. It is characterized by the accumulation of monotypic light chains of proximal tubular epithelial cells in crystalline or non-crystalline form, resulting in proximal tubular dysfunction, which is often manifested as Fanconi syndrome (FS). The excessive light chains deposited in the proximal and distal tubules exhibit distinct characteristics, rendering the coexistence of LCCN and LCPT a particularly rare occurrence. In this case, we encountered an unusual presentation of λ light chain-restricted non-crystalline LCPT with LCCN, which manifested as AKI. This case was easily missed due to the unusual presentation and the hidden pathological morphology. To our knowledge, only 14 similar cases have been reported and we present a literature review and summary of these cases to further the understanding of the disease and improve the diagnostic accuracy.

## Case presentation

The patient, a 49-year-old male, presented with fatigue, lethargy, poor diet and thirst 40 days ago. He was initially treated with medications for “dyspepsia” at a local clinic (unknown). With the above symptoms being persisted, the patient sought further medical attention at another hospital 10 days ago. At this time, he did not present with edema, normal urination, chest tightness, shortness of breath, dizziness or headache. Urinalysis showed both protein and occult blood were negative. The renal function analysis showed a urea level of 22.94mmo1/L, and a creatinine level of 644umo1/ L. The peripheral blood tests revealed white blood cell levels of 3.04 × 10^9^/L, red blood cell levels of 6.72 × 10^12^/L, and hemoglobin of 96 g/L. The patient was diagnosed with “renal failure” and treated with symptomatic medication (unknown) and one hemodialysis procedure, but the symptoms did not get significantly relieved. He was admitted to our hospital for further diagnosis and treatment. Upon admission, the patient had poor mental performance, poor appetite, the weight loss of 7 kg, stable vital signs, and normal bowel movements. The patient had no preexisting chronic conditions, denied a history of drug and food allergies, and had no history of trauma, blood transfusion, or poisoning. The renal function test showed a creatinine level of 661.0umol/L, urea nitrogen of 14.4 mmol/L, and carbon dioxide of 33.3 mmol/L. Other indicators showed a total protein concentration of 57.5 g/L, albumin of 36.2 g/L, serum β2 microglobulin of 15.2 mg/L, red blood cell count of 2.66 × 1012/ mL. The patient’s hemoglobin was 81 g/L, triglyceride was 2.15 mmol/L, fibrinogen degradation was 18.34 µg/ml, and D-dimer was 3.823 µg/ml. There were no abnormalities in liver function or electrolytes. The urine analysis yielded negative results for the presence of protein and occult blood. The urinary excretion of amino acids, phosphate and uric acid was within the normal range. Urine microscopy showed no cells or casts. The serum and urine λ light chain levels were increased to 1235 mg/dl and 93.25 mg/dl respectively. The ratio of serum and urine κ to λ free light chain was 0.0022 and 0.0316 respectively. Additionally, serum protein electrophoresis demonstrated an M-spike with monoclonal IgD-λ. The patient was treated with hemodialysis, antioxidants, and renal protection. A kidney color ultrasound revealed normal renal function in both kidneys. Kidney biopsy and bone marrow puncture were subsequently performed.

**Fig. 1:**
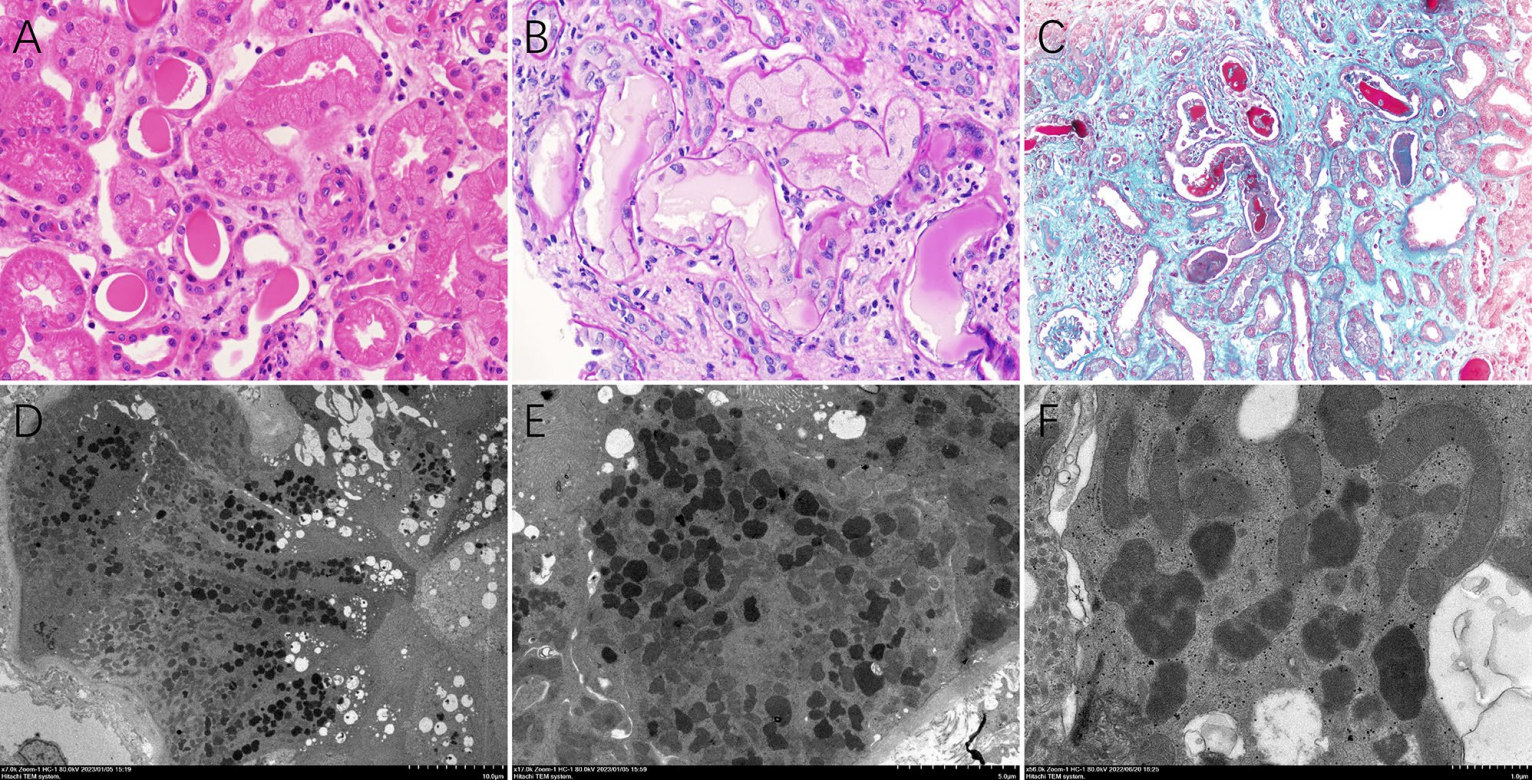
**A**. The prominent injury proximal tubules with coarse cytoplasmic vacuolization and brightly eosinophilic intracytoplasmic inclusions (HE, original magnifcation×400). **B**. The engorged proximal tubular cells with abundant small PAS-negative inclusions and the distal tubule with PAS-negative cast (PAS, original magnifcation×400). **C**. Giant cell reaction around the casts and obstructed distal tubule (Masson, original magnifcation×200). **D** and **E**. The enlarged proximal tubular cytoplasm with numerous atypical lysosomes (electron microscopy, original magnification ×7000 and 28000). **F**. Focally, the prominent intracytoplasmic electron dense inclusions in proximal tubular epithelial cell lysosomes without crystalline (electron microscopy, original magnification ×56000)

**Fig. 2:**
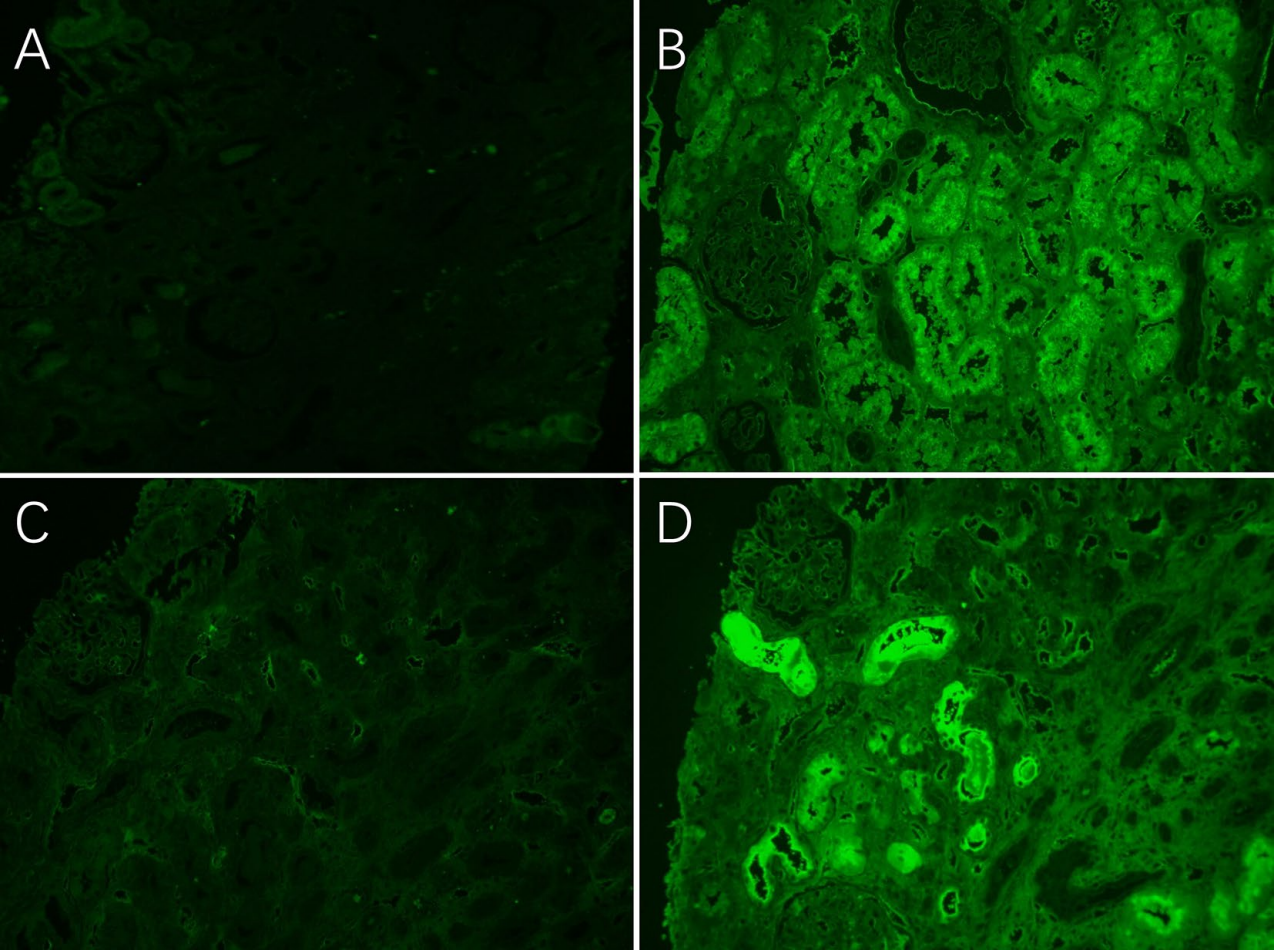
The intense granular negative for κ light chains (**A** and **C**) but positive for λ light chains (**B** and **D**) in the proximal tubular cells and the distal tubular cast (immunofluorescence, original magnification ×200)

The bone marrow puncture results indicated the presence of MM with 30.5% primitive naive plasma cells. Light microscopy of the renal biopsy showed thirty glomeruli, all of which exhibited no notable pathological changes, with only a few mild segmental hyperplasia in the mesangial stroma. However, the renal tubule exhibited diffuse injury, interstitial fibrosis with edema, and notable lymphocyte and histiocytic infiltration. In particular, some of the proximal tubules had diffuse loss of brush borders, coarse cytoplasmic vacuolization and bright eosinophilic intracytoplasmic inclusions, as well as sloughing of cytoplasm into tubular lumina. (Fig. 1A). In select proximal tubules, the cytoplasm was observed to be PAS-negative, and in the distal tubular lumen, several PAS-negative casts with a rigid appearance were noted (Fig. 1B). A giant cell reaction was observed around the casts and obstructed distal tubules (Fig. 1C). Electron microscopy revealed that the proximal tubular cytoplasm was enlarged with numerous atypical lysosomes (Fig. 1D and E) and prominent intracytoplasmic electron-dense inclusions in proximal tubular epithelial cell lysosomes (Fig. 1F). No crystalline inclusions were observed in proximal tubular epithelial cell and the distal tubular casts. By direct IF microscopy, the intracytoplasmic inclusions and cast were negative for κ light chains (Fig. 2A and C) and positive for λ light chains (Fig. 2B and D) by repeating the latter after digestion with protease of the paraffin sections. Combined with clinical and bone marrow puncture findings, we reached the overall pathological diagnosis of LCPT with LCCN secondary to IgD-λ MM.( Supplement Figures (S1, S2)

The patient was then treated with an eight-cycle induction chemotherapy comprising a combination of bortezomib, lenalidomide and dexamethasone in conjunction with regular dialysis therapy. With this regimen, a repeat bone marrow biopsy was conducted 9 months after the initiation of treatment, which showed normocellular marrow with no evidence of plasma cell neoplasm.

One year later, the patient’s renal function improved to his baseline level, with serum creatinine of 1.6 mg/dl and the free light chain κ/λ ratio reduced to 26. Currently, the patient is still in a state of partial remission, and is waiting to proceed with autologous hematopoietic stem cells mobilization and harvest, followed by high-dose chemotherapy with autologous transplantation.

## Discussion and conclusion

Monoclonal immunoglobulin light chain deposition with the kidney tubular damage is a common complication of multiple myeloma. One of the most frequent outcomes is severe kidney failure resulting from Bence-Jones proteinuria [1]. LCPT and LCCN are two pathological forms of renal tubule injury, which rarely occur simultaneously. Various microscopic and ultrastructural changes in the kidney tissue provide a strong evidence for the diagnosis of monoclonal immunoglobulin nephropathy in the patients with multiple myeloma. Without such evidence, the diagnosis is easily overlooked. Here, we present a rare case of combined non-crystalline LCPT and LCCN with λ light chain-restricted and review the previously published cases. A clinicopathological analysis of these cases will further improve the understanding of a rare combination of LCCN with LCPT in multiple myeloma, and will help to reduce the number of missed diagnoses.

At present, the pathological mechanism of LCPT remains unclear. It is postulated that the abnormal molecular structure, changes in physicochemical properties and changes in the quantity of monoclonal light chains may be related to the pathogenesis of LCPT. There is considerable heterogeneity in the induction of renal tubular injury by monoclonal free light chains, and the toxicity of monoclonal free light chains (FLCs) is also highly variable [2]. Under normal conditions, a small number of monoclonal free light chains filtered by glomerular filtration are reabsorbed via the megalin/cubilin scavenger receptor on the apical surface of the proximal tubule, endocytosed and transported to the lysosomes for degradation [2]. In patients with MM, excessive FLCs exceed the reabsorption capacity of the proximal tubules and are unable to be degraded by lysosomal enzymes, resulting in crystallisation, or only manifested as an enlargement of the renal tubular epithelial cells with numerous atypical lysosomes but no crystallisation [3]. Studies have shown that a large number of light chains can cause cellular stress and inflammation, but only a few light chains with variable region sequences can cause tubulopathy, either with or without FS. The light chain responsible for FS belongs to the v-κ1 subgroup, which is derived from two V-region mutant sequences (V-κ1–33 and V-κ1–39), the presence of LCO2/O12 sequences and non-polar amino acid residues in the complementary decision region. This results in intracellular crystal formation due to resistance to protein-degrading enzymes. While light chains with the V region of the LCO8/O18 sequence do not typically form crystals, they can directly induce toxicity in the proximal tubules, leading to FS and proximal tubule dysfunction [4, 5]. To form a tubular pattern leading to LCCN, FLCs must reach the distal tubule and bind THP. Studies have shown that FLCs associated with LCPT can aggregate and form crystals in renal tubular epithelial cells, but tend not to bind THP, which may be related to the chemical nature of FLCs [3]. As a result of these properties of FLCs, the two pathological conditions of LCPT and LCCN rarely occur simultaneously. This implies that only if FLCs have an affinity for resistance to both lysosomal enzyme degradation and THP binding can they cause both pathological conditions to occur simultaneously [6]. The case we report was diagnosed with MM and renal biopsy confirmed the rare diagnosis of λ light chain restricted non-crystalline LCPT with LCCN. Notably, neither of these conditions showed the presence of crystals, and the clinical manifestations were AKI rather than FS. This pathological change is completely different from the common classical pathological manifestations, making it more challenging for clinicians to match clinical manifestations with pathological changes. In conclusion, the nephropathy caused by MM can be easily overlooked in the absence of sufficient clinicopathological experience, which may subsequently impact the management of MM-related kidney damage.

To date, a total of 15 cases of LCPT with LCCN have been reported, including the cases we reported. Of these, eight cases had κ light chain restriction and 7 cases had λ light chain restriction (Table 1). Seven cases of crystallization were observed in proximal tubular epithelial cells or casts, with six cases involving κ light chains and one case involving λ light chains. Notably, the only case of crystallization of λ light chain was limited to casts, and no similar crystallization was observed in proximal tubular epithelial cells. From this, it can be observed that when both pathological conditions are present simultaneously, κ light chain restriction is more likely to form crystals, which is consistent with the classical pathological manifestation of κ-light chain restriction LCPT, while λ light chain is significantly more difficult to form crystals. This may be related to the chemical properties of the light chains themselves. Clinically, all patients were diagnosed with MM, and FS was only found in patients with κ light chain restriction and not in patients with λ light chain.

**Table 1:** Cases showing light chain proximal tubulopathy(LCPT)with myeloma cast nephropathy (LCCN)

Year / Authors	Case number	Age/gender	Initial presentation	FS	MM	Crystals	Light chains
2000 Messiaen et al.	8	57/F	Bone pain, AKI	Yes	Yes	PTEC	κ
	9	65/F	Bone pain, AKI	Yes	Yes	None	κ
	10	74/F	Fatigue, weight loss	Yes	Yes	None	κ
2012 Sharma et al.	8	58/M	Proteinuria, AKI	No	Yes	None	λ
	9	63/M	Proteinuria, AKI	No	Yes	None	λ
2015 Godwa et al.	1	60/F	Fatigue, proteinuria, AKI	No	Yes	None	λ
2016 Gallan et al.	1	59/F	Fatigue, proteinuria, AKI	No	Yes	Casts	λ
2017 Wu et al.	1	48/M	Anorexia, AKI	No	Yes	PTEC	κ
2017 Koratala et al.	1	69/M	Fatigue, anemia, AKI	No	Yes	PTEC	κ
2018 Kishi et al.	1	78/F	anemia, AKI	No	Yes	None	λ
2020 Chopra et al.	1	64/M	FS, AKI	Yes	Yes	PTEC	κ
2020 Lerner et al.	1	66/F	AKI	No	Yes	PTEC and Casts	κ
2020 Matthai et al.	1	48/F	NS, anemia, AKI	No	Yes	PTEC	κ
2020 Lin et al.	1	44/F	anemia, AKI,	No	Yes	None	λ
Our case	1	49/M	Fatigue, anemia, AKI	No	Yes	None	λ

FS, Fanconi syndrome; MM, multiple myeloma; PTEC, Proximal tubular epithelial cells; AKI, acute kidney injury; NS, nephrotic syndrome; F, female; M, male

In summary, this is a rare case of LCPT with LCCN secondary to MM in a patient with a severe clinical presentation. The pathological manifestations on renal biopsy are significant for the patient with haematological malignancies. In addition, λ light chain-restricted non-crystalline LCPT with LCCN is a rare renal manifestation in patients with IgD-λ MM. It is crucial for pathologists to comprehensively understand these diseases and their pathological manifestations.

## Electronic supplementary material

Below is the link to the electronic supplementary material.

Supplementary Material 1

Supplementary Material 2

## Data Availability

Data is provided within the manuscript or supplementary information files.

## References

[1] Stokes MB, Valeri AM, Herlitz L, Khan AM, Siegel DS, Markowitz GS, D’Agati VD. Light Chain Proximal Tubulopathy: clinical and pathologic characteristics in the Modern Treatment Era. J Am Soc Nephrol. 2016;27(5):1555–65. 26374607 10.1681/ASN.2015020185 PMC4849818

[2] Sirac C, Batuman V, Sanders PW. The proximal tubule toxicity of Immunoglobulin Light Chains. Kidney Int Rep. 2021;6(5):1225–31. 34013100 10.1016/j.ekir.2021.02.026 PMC8116766

[3] Hogan JJ, Alexander MP, Leung N. Dysproteinemia and the kidney: core curriculum 2019. Am J Kidney Dis. 2019;74(6):822–36. 31331759 10.1053/j.ajkd.2019.04.029

[4] Luciani A, Sirac C, Terryn S, Javaugue V, Prange JA, Bender S, Bonaud A, Cogne M, Aucouturier P, Ronco P, et al. Impaired lysosomal function underlies Monoclonal Light Chain-Associated Renal Fanconi Syndrome. J Am Soc Nephrol. 2016;27(7):2049–61. 26614382 10.1681/ASN.2015050581 PMC4926980

[5] Sethi S, Rajkumar SV, D’Agati VD. The complexity and heterogeneity of Monoclonal Immunoglobulin-Associated Renal diseases. J Am Soc Nephrol. 2018;29(7):1810–23. 29703839 10.1681/ASN.2017121319 PMC6050917

[6] Leboulleux M, Lelongt B, Mougenot B, Touchard G, Makdassi R, Rocca A, Noel LH, Ronco PM, Aucouturier P. Protease resistance and binding of ig light chains in myeloma-associated tubulopathies. Kidney Int. 1995;48(1):72–9. 7564094 10.1038/ki.1995.269

